# Detection of Generalized Tonic–Clonic Seizures in Dogs With a Seizure Detection System Established Using Acceleration Data and the Mahalanobis Distance: A Preliminary Study

**DOI:** 10.3389/fvets.2022.848604

**Published:** 2022-04-28

**Authors:** Junya Hirashima, Miyoko Saito, Tsukasa Kuriyama, Taketo Akamatsu, Minoru Yokomori

**Affiliations:** Laboratory of Small Animal Surgery (Neurology), School of Veterinary Medicine, Azabu University, Sagamihara, Japan

**Keywords:** canine, dog, epilepsy, Mahalanobis distance, seizure detection, wearable device, accelerometer

## Abstract

Caregivers of dogs with epilepsy experience severe stress due to unpredictable seizures. Hence, they feel the need for a better management strategy. A seizure detection system (SDS), which can identify seizures and provide notifications to caregivers immediately, is required to address this issue. The current study aimed to establish a wearable automatic SDS using acceleration data and the Mahalanobis distance and to preliminarily investigate its feasibility among dogs. A generalized tonic–clonic seizure (GTCS) was targeted because it is the most common type of seizure and can have serious consequences (i.e., status epilepticus). This study comprised three phases. First, the reference datasets of epileptic and non-epileptic activities were established using acceleration data of GTCSs in 3 dogs and daily activities in 27 dogs. Second, the GTCS-detecting algorithm was created using the reference datasets and was validated using other acceleration data of GTCSs in 4 epileptic dogs and daily activities in 27 dogs. Third, a feasibility test of the SDS prototype was performed in three dogs with epilepsy. The algorithm was effective in identifying all acceleration data of GTCSs as seizures and all acceleration data of daily activities as non-seizure activities. Dogs with epilepsy were monitored with the prototype for 48–72 h, and three GTCSs were identified. The prototype detected all GTCSs accurately. A false positive finding was not obtained unless the accelerometer was displaced. Hence, a method that can detect epileptic seizures, particularly GTCSs, was established. Nevertheless, further large-scale studies must be conducted before the method can be commercialized.

## Introduction

Epilepsy is a chronic brain disorder that causes epileptic seizures. It can impair the quality of life (QOL) of dogs and their owners due to various reasons (1–5). In particular, epileptic seizures are unpredictable in nature, and prolonged seizure (i.e., status epilepticus) can be life-threatening. Thus, anxiety correlated with sudden seizures causes severe stress among owners, and this may reduce QOL (2, 4). However, most owners find it challenging to monitor dogs with epilepsy, and this notion is true for veterinarians and clinical staff who manage dogs with epilepsy in the hospital. A seizure detection system (SDS) that can be used for continuous monitoring and the detection and notification of seizures can reduce burden among owners and veterinarians. Based on a recent study, the owners of dogs with epilepsy want to use a seizure detection device (6). Such a system can accurately identify seizure frequency, which is important for the medical/surgical treatment of epilepsy.

An accelerometer is used to evaluate the behavior and activity of dogs (7–12). Moreover, it is applied for not only motion analysis but also for medical research, such as the evaluation of therapeutic effects of treatment among dogs with osteoarthritis and the assessment of pruritus degree due to dermatosis (13, 14). We hypothesized that epileptic seizures in dogs can be detected using acceleration data.

The Mahalanobis distance is a type of discriminant analysis, which determines the distance between the test dataset and several reference datasets. The test dataset belongs to the same category as the reference dataset with the shortest distance. Therefore, the Mahalanobis distance is used for outlier detection in the engineering field (15, 16). One study investigated whether the behaviors of cows, including resting, ruminating, and eating, can be identified using acceleration data and the Mahalanobis distance (17). In this study, the validity of behavior identification was 95%. We considered epileptic seizures as one motion and hypothesized that seizures can be differentiated from other motions and detected correctly.

The current research aimed to develop an automatic SDS using acceleration data and the Mahalanobis distance and to preliminarily investigate its feasibility among dogs. Moreover, a generalized tonic–clonic seizure (GTCS) was targeted because it is the most common type of seizures (18–20) and can cause serious complications (i.e., status epilepticus).

## Methods

### Animals

In total, 24 laboratory dogs, including 7 dogs with epilepsy (Dogs A–G) and 17 healthy dogs, 2 patient dogs (Dogs 1 and 2) with epilepsy that were hospitalized in Azabu University Veterinary Teaching Hospital, and 11 pet dogs were included in this study. Supplementary Tables 1–3 depict the information of these dogs. Seven laboratory dogs with epilepsy (Dogs A–G) were diagnosed with idiopathic epilepsy (IE), which was made using the International Veterinary Epilepsy Task Force (IVETF) criteria (21). The seizure types included GTCSs and/or focal motor seizures that evolved to GTCSs. Dog 1 was diagnosed with IE based on tier III level of the IVETF consensus proposal (21). Dog 2 had structural epilepsy caused by brain tumor, which was diagnosed via magnetic resonance imaging. Both the patient dogs had GTCSs. In total, 17 laboratory dogs and 11 pet dogs had good general condition and did not experience seizures. The owners of 11 pet dogs were acquaintances of the investigators, and they volunteered to join in this study. This study was approved by the Institutional Research Committee at Azabu University, and all procedures were conducted following the guidelines approved by this committee (approval number: 110606, 200330-1, 201007-4).

### Accelerometer

**Figure 1:**
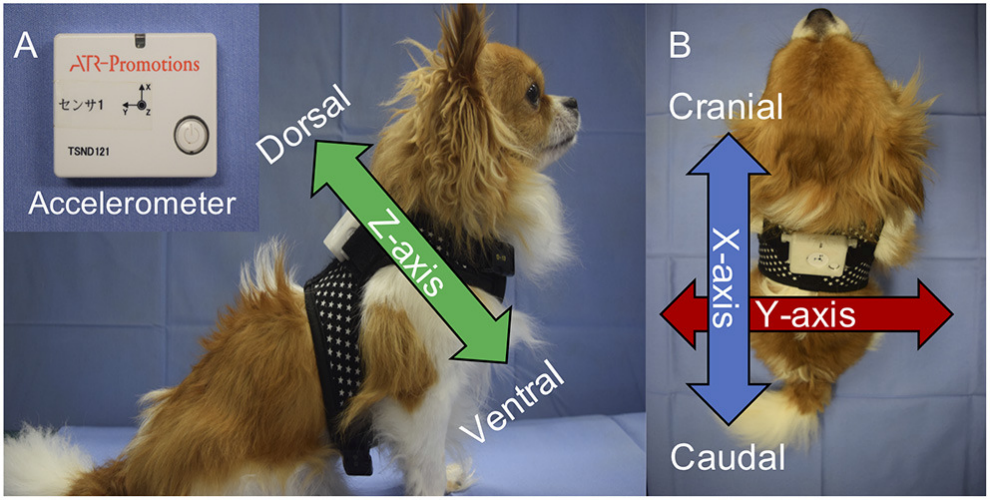
The accelerometer (TSND121, ATR-Promotions) and its position on the dogs. The width of the device was 37 mm; height, 46 mm; depth, 12 mm; and weight, 22 g **(A)**. The accelerometer was placed on the interscapular region with the harness **(A,B)**. The X-axis was craniocaudal; the Y-axis, lateral; and the Z-axis, dorsoventral **(A,B)**.

A wireless three-axis accelerometer (TSND121, ATR-Promotions, Japan) was used to obtain acceleration data (Figure 1A). The sensor was programmed at a sampling frequency of 50 Hz and at an acceleration range of ± 8 g. The accelerometer was tied to a harness with plastic clamping bands. This harness was worn by the dogs during all experiments. The accelerometer was positioned at the interscapular region. Hence, the X-axis was in the craniocaudal direction, the Y-axis in the lateral direction, and the Z-axis in the dorsoventral direction (Figures 1A,B). To prevent rotation of the harness and accelerometer, the former was selected according to body size, and it fitted well. Acceleration data were recorded and saved on a computer using the accelerometer software (Sensor Controller, ATR-promotions, Japan). The recorded acceleration data were exported into Microsoft Excel (Microsoft Corp., Redmond, WA, USA) and were processed.

### Experimental Protocol

**Figure 2:**
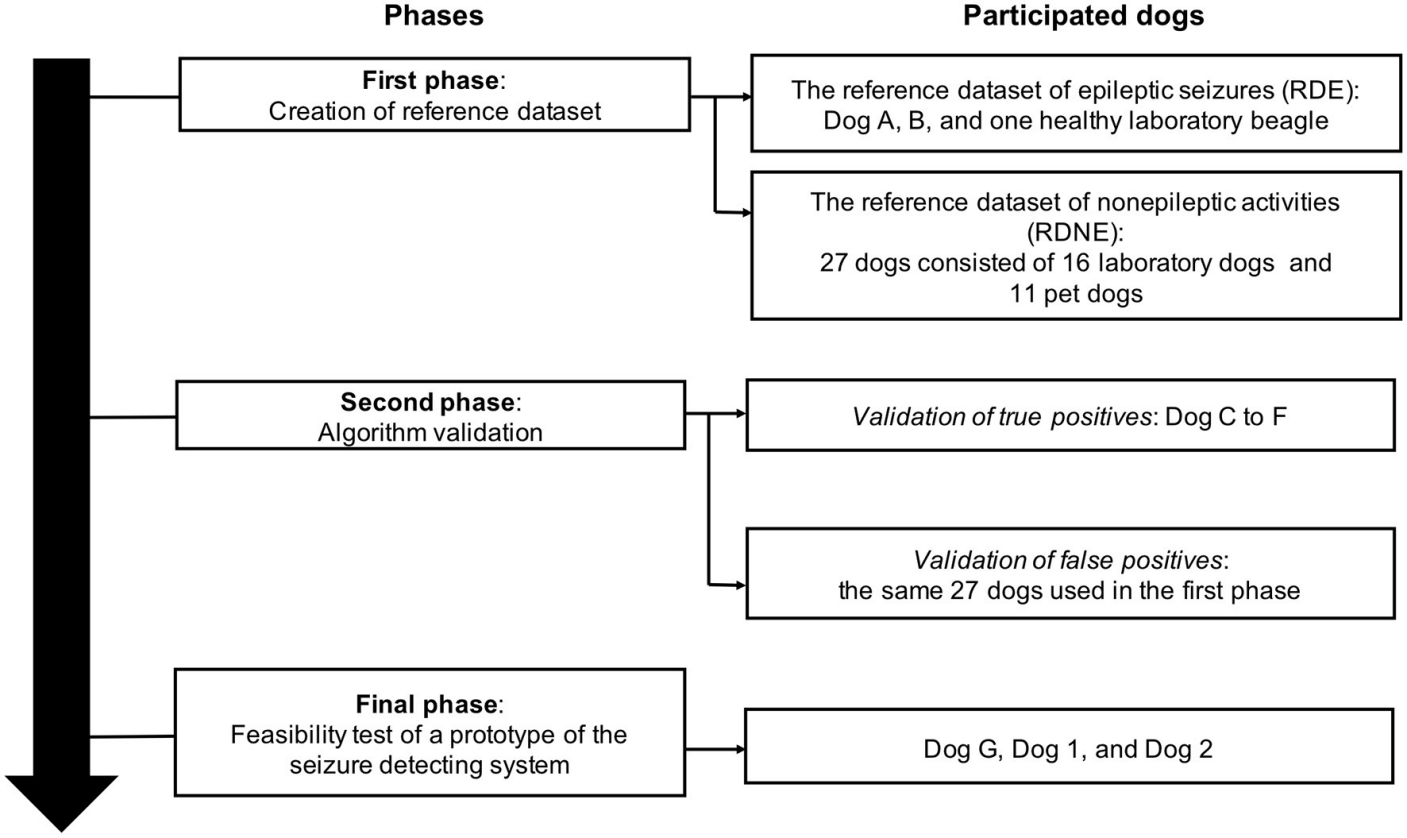
The flowchart of this study, which comprised three phases: creation of the reference datasets in the first phase, validation of the algorithm in the second phase, and feasibility testing of the prototype of the seizure detection system in the final phase.

Figure 2 shows the flowchart of this study and information about the dogs that participated in each phase of the experiment. This study comprised three phases. In the first phase, the reference dataset of epileptic seizures (RDE) and the reference dataset of non-epileptic activities (RDNE) were established using the acceleration data of GTCSs and daily activities, respectively. In the second phase, the GTCS-detecting algorithm was established and validated using the RDE and RDNE. Finally, a feasibility test of the prototype of SDS that implemented the algorithm was performed on dogs with epilepsy.

### First Phase: Creation of Reference Dataset

To establish the RDE, GTCSs acceleration data were obtained from three beagles (Dogs A and B and one healthy laboratory dog). However, the frequency of seizure in Dogs A and B was extremely low (once to twice a year). Thus, to collect acceleration data of GTCSs, bemegride (Medibal; Mitsubishi Tanabe Pharma Corporation, Osaka, Japan), a seizure inducer, was used in the three dogs (22). Bemegride 20 mg was administered intravenously for over 30 s to induce seizure based on the methodology in a previous study conducted by the authors (23). Although bemegride induces seizures, it had no other undesirable adverse effects in dogs (23). If a seizure occurred, bemegride was discontinued immediately, even before the dosage reached 20 mg. Immediately after the seizure ceased or 5 min had elapsed after seizure onset, we administered intravenous diazepam (0.5 mg/kg; Horizon Injection 10 mg; Maruishi Pharmaceutical, Osaka, Japan), followed by phenobarbital (3 mg/kg; Phenobal Injection 100 mg; Fujinaga Pharma Corporation, Tokyo, Japan), which are bemegride antagonists (24, 25). If the seizure did not cease, two doses of diazepam were administered additionally. In case the seizure continued, standard treatment for status epilepticus was started.

After seizure cessation, the dogs were monitored constantly for 24 h. Moreover, oral levetiracetam was administered if seizures occurred during observation. The dogs were video-recorded from the start of bemegride administration to seizure cessation. A radio clock was used to record the videos to validate the time of events. The times of the radio clock and the accelerometer were synchronized before the start of each experiment. Only acceleration data recorded during the tonic–clonic phase were used for the RDE creation. The average of the GTCS acceleration data was obtained, and their variance-covariance matrix was calculated. The average and the variance-covariance matrix comprised the RDE.

To create the RDNE, daily activity acceleration data were collected from 27 dogs (16 healthy laboratory dogs and 11 pet dogs). The acceleration and video data of different daily activities (non-epileptic activities) were collected using the following method. The laboratory dogs stayed in a test room, which was a 2.8 × 4.1-meter space, and they moved freely during data recording. The radio clock, which synchronized its time with the accelerometer, was video-recorded. The data of pet dogs were recorded in their house or outside, and they moved freely and performed their daily activities. The investigator followed the dogs and recorded the video. The times of the accelerometer and the clock in the video camera were synchronized. In both laboratory and pet dogs, data recording was ended if a dog stopped moving spontaneously or 60 min had passed. This experiment was performed once on each dog. The daily activities of the dogs were classified into several movements such as walking and sitting. The dataset of each movement was created by extracting consecutive 9-s acceleration data from dogs that performed the movement. For example, if 10 dogs walked, the total 90-s acceleration data comprised the dataset of walking. For movements that did not last for 9 s, the 9-s acceleration data were created by combining data obtained at different timings. Nine seconds was used because at least nine data were required to calculate the variance-covariance matrix. The average of the dataset of all movements was obtained, and their variance-covariance matrix was calculated. The average and the variance-covariance matrix comprised the RDNE.

### Second Phase: Algorithm Validation

#### Algorithm Procedure

In the second phase, we created the GTCSs detection algorithm^*^ using data generated in the first phase. The algorithm procedure was as follows: First, the resultant force was calculated using three-axis acceleration data. The average and coefficient variation of the three-axis and the resultant force for each second were calculated. Thus, eight parameters (i.e., the average and coefficient variation of the X-, Y-, and Z-axis and the resultant force) existed in each second (Supplementary Figure 1). A test dataset comprised the 9-s epoch of the eight parameters (Supplementary Figure 1). Subsequently, the Mahalanobis distance between the test dataset and the two pre-generated reference datasets; the RDE and RDNE, was calculated. If the Mahalanobis distance of the test dataset obtained from a dog was shorter in the RDE than in the RDNE, the test dataset belonged to the RDE. Hence, the dog had epileptic seizures. However, if the Mahalanobis distance of the test dataset recorded from a dog was shorter in the RDNE than in the RDE, the test dataset belonged to the RDNE. Therefore, the dog did not present with seizures. The Mahalanobis distance calculation was performed in Microsoft Excel by programming its formula into Excel VBA (Microsoft Corp., Redmond, WA, the USA).

#### Algorithm Validation

Four laboratory dogs (Dogs C–F) were used to validate true positive findings. In the second phase, the presence of true positive results indicated that the algorithm was accurate in identifying that the GTCSs test dataset belonged to the RDE. The acceleration data of GTCSs in these dogs were collected using the following method. Dogs C and D were treated with anti-seizure drugs (ASDs) for epilepsy. Treatment with ASDs was discontinued temporarily, and the dogs were monitored continuously until a seizure occurred. Immediately after the seizure ceased or 5 min had elapsed after the seizure onset, diazepam (0.5 mg/kg; Horizon Injection 10 mg; Maruishi Pharmaceutical, Osaka, Japan), followed by phenobarbital (3 mg/kg; Phenobal Injection 100 mg; Fujinaga Pharma Corporation, Tokyo, Japan), was administered intravenously. If the seizures did not cease, the same treatment as in phase 1 was provided. Acceleration data were collected throughout this experimental period. After the seizure cessation, the dogs were assessed continuously for 24 h. Treatment with maintenance ASDs was restarted. Additionally, oral levetiracetam (Ekeppra 500 mg; Otsuka Pharmaceutical, Tokyo, Japan) was administered at a dose of 20 mg/kg every 8 h for 3 days to prevent the development of another seizure. Dogs C and D were video-recorded from the time when ASDs were withdrawn to the time when the seizure occurred and then ceased. In case Dog C or D did not develop a seizure 36 h after ASD withdrawal, bemegride induction was started. Since the frequency of seizures was extremely low in Dogs E and F, bemegride was used to induce a GTCS in the same manner as Dogs A and B. The 9-s epoch of the eight parameters immediately after the GTCS onset in each dog was extracted as the GTCSs test dataset. The GTCSs test datasets were processed with the algorithm.

The acceleration data of daily activities obtained from 27 dogs (16 healthy laboratory dogs and 11 pet dogs) in the first phase were used to validate false positive findings. In the second phase, the presence of false positive results indicated that the algorithm was not accurate in identifying that the non-epileptic activity test dataset belonged to the RDNE. All recorded acceleration data from each dog were combined as continuous data. This combined data was divided into 9-s epochs in the non-epileptic activity test dataset. The test datasets were created repeatedly by shifting the beginning of the 9-s epoch every 1 s (Supplementary Figure 2). The non-epileptic activity test datasets were processed with the algorithm.

### Final Phase: Feasibility Test of a Prototype of the SDS

A prototype of SDS, which implemented our algorithm, was established, and the feasibility test of the SDS was performed on three dogs with epilepsy. This system comprised a wireless three-axis accelerometer, a monitoring device that used the seizure algorithm created in the second phase, and a smartphone (Supplementary Figure 3). The sampling frequency and the acceleration range of the accelerometer were 50 Hz and ±8 g, respectively. The accelerometer linked to the monitoring device via Bluetooth was placed on the midline of the dorsum of the dog using a harness or custom-made jacket. The monitoring device provided seizure notifications to the smartphone, and it detects GTCSs real-time. Dog G and Dogs 1 and 2 with epilepsy were monitored with the system for 72 and 48 h, respectively. These dogs were continuously monitored by the investigators during the feasibility test to determine whether the prototype detected seizures accurately (true positive). Moreover, the occurrence of false positive findings (detecting a seizure even though a dog did not actually have one) and false negative findings (failure to detect a seizure in a dog that actually experienced one) was assessed by the investigators.

## Results

### First Phase: Creation of Reference Dataset

Regarding the RDE, three beagles presented with GTCSs immediately after starting treatment with bemegride. However, it ceased spontaneously within 5 min. The dogs were managed with diazepam and phenobarbital in accordance with the abovementioned protocol. No additional seizures were observed. Hence, the three GTCSs acceleration data comprised the RDE.

Regarding the RDNE, the median acceleration recording time in 27 dogs was 21 (range: 8–60) mins. The total recording time was 696 min (41,760 s). Fifteen different types of movements in dogs confirmed in the video, such as walking and lying, were selected as daily activities (Table 1). Table 1 presents the number of dogs that performed each movement during data recording. For example, 23 dogs performed walking. Hence, the total 1,215-s acceleration dataset obtained from 15 movements comprised the RDNE.

**Table 1:** Fifteen movements comprising the reference dataset of non-epileptic activities and the number of dogs comprising the dataset of each activity.

**Movements**	**Number of dogs**
Walking	23
Standing	15
Shaking	24
Drinking	7
Running	4
Jumping on a sofa	3
Jumping off a sofa	3
Lying on the stomach	12
Lying on the side	5
Scratching	4
Playing with a toy	3
Being stroked	8
Sitting	14
Changing position from lying on their stomach to lying on their side	5
Changing position from lying on their side to lying on their stomach	5

*For instance, acceleration data during walking in 23 dogs comprised the dataset of walking. Thus, the walking dataset comprises 207-s acceleration data (9 s for each dog). The cumulative total was 135 dogs; therefore, the 1,215-s acceleration data comprised the reference dataset of non-epileptic activities*.

### Second Phase: Algorithm Validation

In total, there were four GTCSs recorded (Supplementary Video 1). Dog C experienced a spontaneous GTCS after withdrawal of ASDs. In Dogs D, E, and F, it was induced by bemegride. Hence, their acceleration data were used for validation. Three drug-induced GTCSs ceased spontaneously within 5 min. Four GTCSs test datasets were created. All datasets had shorter Mahalanobis distances in the RDE than in the RDNE (Table 2). Therefore, they were classified accurately. That is, there were only true positive and no false negative results.

**Table 2:** The Mahalanobis distance between the GTCS test dataset and the reference dataset.

**Dogs**	**Type of seizures**	**Number of recorded seizures**	**Distance to the RDE**	**Distance to the RDNE**	**Reference data to which the test dataset belongs to**
C	GTCS	1 (spontaneous)	5.3	5.38	RDE
D	GTCS	1 (drug-induced)	3.43	6.2	RDE
E	GTCS	1 (drug-induced)	4.75	5.32	RDE
F	GTCS	1 (drug-induced)	4.77	5.43	RDE

*All GTCS test datasets recorded from each dog had a shorter distance to the reference dataset of epileptic seizures (RDE) than the reference dataset of non-epileptic activities (RDNE). Therefore, all GTCS test datasets belonging to the RDE were identified accurately*.

As 1,215 of 41,760-s acceleration data were used for creating the RDNE, the remaining 40,545-s daily activity acceleration data were used for validation. Thus, 40,537 non-epileptic activity 9-s epoch test datasets were established. All non-epileptic activity test datasets had shorter Mahalanobis distances in the RDNE than in the RDE. Therefore, all non-epileptic activity test datasets were classified accurately; that is, there were no false positive findings.

### Final Phase: Feasibility Test of a Prototype of the SDS

The three epileptic dogs were under the SDS monitoring for 168 h. Dog G had three seizures during monitoring. The seizures were focal motor epileptic seizures that evolved into GTCSs. The prototype detected all three seizures accurately, and the seizure notification was sent to the smartphone. Thus, there were three true positive findings. Two patient dogs did not have seizures during monitoring, and a false negative finding was not obtained. In one patient dog, a false positive finding was observed 6 times when the dog was walking around in the cage. However, the accelerometer was displaced on the harness laterally during these times. Other than that, there were no false positive findings in all three dogs.

## Discussion

This preliminary study investigated whether GTCSs can be detected using acceleration data and the Mahalanobis distance, which is the initial step toward the development of the SDS. Via algorithm validation and feasibility testing of the SDS prototype, our algorithm was accurate in identifying all four acceleration data acquired from each GTCS as seizures. Further, the prototype of the SDS was effective in detecting all three seizures in one dog. False positive findings were not obtained except in a case in which the accelerometer was displaced in 40,545-s daily activity acceleration data in 27 dogs and during the 168-h monitoring period in three dogs with epilepsy. Therefore, the development of the SDS with our algorithm should be further advanced.

Several studies aimed to detect or predict epileptic seizures in humans (26–32). If the target is limited to GTCSs, the sensitivity of seizure detection using an accelerometer is 79–91% (26–28). Interestingly, our canine study revealed accurate seizure detection. In humans, GTCSs, particularly clonic seizures, is characterized by a burst-like acceleration pattern caused by intense movement (31). Dogs with clonic seizures had an intense movement. Therefore, GTCSs were likely detected using an accelerometer in dogs. The current study supports the hypotheses that seizures can be differentiated from other motions and GTCSs can be detected accurately.

A recent study using an accelerometer on a collar for detecting seizures in dogs revealed that generalized seizures could be detected with an accelerometer (33), which is consistent with our results. However, the overall sensitivity of identifying generalized seizures in this previous research was ~20%. Although the sample size was small and ~50% of dogs experienced drug-induced seizures (3 bemegride-induced and 1 spontaneous in the second phase and 3 spontaneous in the final phase) in this study, our algorithm detected all seven GTCSs without any false positive findings if the system was used properly. The prior study used machine learning (random forest classifier) to detect seizures. In this report, the Mahalanobis distance was utilized. Machine learning has been applied in human seizure detection studies (26–32). Humans take different postures and perform complex movements. Thus, machine learning that detects patterns or rules from large datasets could be used as seizure-detecting methods. Alternatively, the postures and movements of dogs are relatively uncomplicated compared with those of humans. Thus, a simpler algorithm can detect seizures in dogs. Using the Mahalanobis distance, we created a simple two-choice algorithm (seizures or not seizures). The conciseness of this algorithm is likely one of the reasons why our method could detect convulsive seizures more accurately.

Our method requires a test dataset with 9-s epoch. We preliminarily assessed the algorithm using datasets with different time durations. If a 3-s or 6-s epoch was used, GTCSs were detected accurately. However, false positive findings were obtained several times. That is, the algorithm determined that non-epileptic activity test dataset belongs to the RDE. Meanwhile, in a 9-s epoch, there were few false positive findings, as shown in the current study. At least 9-s data were required to differentiate seizures from non-seizure activities. Generalized tonic–clonic seizures lasts for more than 9 s in most cases, and rapid treatment is important to prevent status epilepticus. Hence, the algorithm using 9-s data is feasible for practical use.

Our seizure detection algorithm targets GTCSs. Several studies have shown that 68–94% of dogs with epilepsy present with GTCSs (18–20). Therefore, most dogs with epilepsy experience GTCSs. In addition, status epilepticus, which can be life-threatening, often presents with GTCSs. Thus, a device targeting GTCSs is clinically important in veterinary medicine.

The SDS appears to work regardless of dog breed. Whether breeds affected the detection of GTCSs needs to be investigated. The RDE using a single breed of the beagle was first established, and the algorithm was verified with other different breeds. One healthy beagle was included because only two epileptic laboratory beagles were available during the conduct of the first phase of the study. Although the RDE comprised data from three dogs of a single breed, seizures were detected accurately in five different breeds. Further, no false positive finding was confirmed in 10 different breeds. Therefore, our algorithm is likely to be applicable to any dog breeds.

A harness or jacket was used to place the accelerometer on dogs in this study. We believe that a change in the three-axis direction of the accelerometer could affect the detection of epileptic seizures. Moreover, the position of the accelerometer could change due to collar rotation. Thus, a harness or jacket that does not easily rotate was used. Indeed, a false positive result was only obtained in cases in which the accelerometer was displaced in one patient dog using the prototype. Therefore, the accelerometer should be maintained in the correct position to ensure that the seizure detection algorithm is working accurately. However, the three dogs enrolled in the final phase were cage-confined during the monitoring. Thus, false-positive findings may increase in dogs living at home.

The SDS can have several benefits in veterinary medicine. Numerous studies showed that the caregivers of epileptic dogs, particularly those who have less time to supervise the dog directly, experience stress and anxiety due to unpredictable seizures (2, 4, 6). Medical staff may have a similar experience because continuous monitoring is not always easy in some busy clinics. Our algorithms can allow the SDS to send automatic notification to the smart phone of caregivers if a seizure is detected. The person who receives the alert can check the dog's safety remotely by watching the live streaming monitor. Moreover, emergency medications may be administered more readily. Seizure frequency is an indispensable information for the seizure management of individual dogs and epilepsy research (34). However, currently, the assessment of seizure frequency is based on the subjective report of caregivers alone. Previous reports in human medicine showed that patients occasionally underestimate seizure frequency (35, 36). Seizure management and epilepsy research can improve with the SDS use as it can accurately evaluate seizure frequency. For epilepsy surgery, the epileptogenic area should be identified for resection or disconnection. The symptomatogenic zone is an area in the cortex that produces the initial clinical sign if a seizure occurs, and it often becomes important in identifying the epileptogenic area (37, 38). Therefore, the initial clinical sign of a seizure onset must be identified accurately. However, video recording of seizures upon onset is often challenging and missed. Hence, the SDS can resolve this issue as it can record the video tracing back several minutes before a seizure onset.

The current study had some limitations. Firstly, the RDE for our seizure-detecting algorithm was established from drug-induced, not spontaneous, seizures. In some cases, the seizures used in algorithm validation were drug-induced. However, we accurately detected spontaneous seizures with the prototype implementing our algorithm. Thus, the reference dataset generated from the drug-induced seizures acceleration data could suffice if it is used as the reference dataset for spontaneous seizures. Secondly, the validation of false-positive findings in the second phase was conducted using acceleration data obtained in the first phase. Using acceleration data recorded from other dogs for the validation would be ideal although no data overlaps were noted between the RDNE and non-epileptic activity test datasets. Lastly, the specificity and sensitivity of our system have not been evaluated because of the small sample size. Obtaining an adequate number of GTCSs acceleration data without inducing was difficult because seizures are unpredictable and the seizure frequency of the most epileptic laboratory dogs in this study was low (less than once per 3 months). Therefore, bemegride, one of the inducers of EEG abnormalities in humans (39), was used. However, the induction of GTCSs to obtain acceleration data in a sufficient number of dogs for specificity and sensitivity evaluation may have ethical concerns. In some countries, medical induction of seizures would not be approved in dogs. Therefore, although the number of dogs included was limited, it was still sufficient for the pilot study. Nevertheless, further large-scale studies must be conducted to obtain clinical data from dogs with epilepsy using a plurality of the SDS prototypes.

We created a method for detecting epileptic seizures (particularly GTCSs) from other movements using acceleration data and the Mahalanobis distance. The algorithm accurately identified all seven GTCSs (four in the validation of our algorithm and three in the feasibility test of the prototype) without any false positive or negative results. Further, the prototype of the SDS implementing this algorithm was effective in detecting all three GTCSs. Thus, the development of the SDS with our algorithm should be further advanced and validated before it can be commercialized.

## Data Availability Statement

The original contributions presented in the study are included in the article/Supplementary Material, further inquiries can be directed to the corresponding author.

## Ethics Statement

The animal study was reviewed and approved by the Ethics Committee of Azabu University, Japan. Written informed consent was obtained from the owners for the participation of their animals in this study.

## Author Contributions

JH: acquired the data, drafted, and wrote the manuscript. MS: conceived and designed the study, wrote the manuscript, and critically revised the article. TK and TA: acquired the data and provided critical feedback. MY: acquired the data, contributed to the initial design of the study, and provided critical feedback. All authors contributed to the article and approved the submitted version.

## Funding

This study was supported in part by JSPS KAKENHI (Grant No. JP17H01507) and a research fund from the Central System Research Corporation (Grant No. Azabu1439).

## Conflict of Interest

The patent of the seizure detection method and monitoring system in this manuscript has been granted and registered. Japanese Patent No. 6193613. The authors declare that the research was conducted in the absence of any commercial or financial relationships that could be construed as a potential conflict of interest.

## Publisher's Note

All claims expressed in this article are solely those of the authors and do not necessarily represent those of their affiliated organizations, or those of the publisher, the editors and the reviewers. Any product that may be evaluated in this article, or claim that may be made by its manufacturer, is not guaranteed or endorsed by the publisher.

## Abbreviations

RDE: Reference dataset of epileptic seizures
RDNE: Reference dataset of non-epileptic activities
GTCS: Generalized tonic–clonic seizure
SDS: Seizure detection system.
