# Supplementary material for: Detection of Generalized Tonic–Clonic Seizures in Dogs With a Seizure Detection System Established Using Acceleration Data and the Mahalanobis Distance: A Preliminary Study
Source: Front Vet Sci. 2022 Apr 28;9:848604. doi: 10.3389/fvets.2022.848604 (PMC9097225; doi:10.3389/fvets.2022.848604)
Supplement: Supplementary file 4 [file Image_1.pdf]

frontiers

Time	X-axis avg (g)	Y-axis avg (g)	Z-axis avg (g)	Resultant avg (g)	X-axis cv (g)	Y-axis cv (g)	Z-axis cv (g)	Resultant cv (g)
20〇〇/〇〇/〇〇 10:27:00	0.23585667	0.25572115	0.94563264	1.0554526	0.42863384	0.58072174	0.08409472	0.067146495
20〇〇/〇〇/〇〇 10:27:01	0.23982164	0.26510945	0.9422667	1.0546426	0.47810358	0.6263182	0.0922258	0.07586935
20〇〇/〇〇/〇〇 10:27:02	0.22422282	0.26760256	0.9432904	1.0540118	0.5368596	0.6315607	0.09436157	0.07830152
20〇〇/〇〇/〇〇 10:27:03	0.22201782	0.26788983	0.94498485	1.0550516	0.49430355	0.59799325	0.0875614	0.07175192
20〇〇/〇〇/〇〇 10:27:04	0.189128	0.26096818	0.9555981	1.0544344	0.46688333	0.49996412	0.07735945	0.06183452
20〇〇/〇〇/〇〇 10:27:05	0.17444256	0.2227722	0.9676049	1.0505434	0.37277424	0.4631078	0.05986796	0.047089994
20〇〇/〇〇/〇〇 10:27:06	0.16980699	0.15381253	0.9947251	1.0487041	0.3324536	0.45870128	0.04630764	0.038756315
20〇〇/〇〇/〇〇 10:27:07	0.16140653	0.09402873	1.0179272	1.0486298	0.34499964	0.4885503	0.03693672	0.034193344
20〇〇/〇〇/〇〇 10:27:08	0.13863334	0.06506795	1.0285888	1.0485132	0.40688527	0.48714215	0.03098419	0.03054409
20〇〇/〇〇/〇〇 10:27:09	0.09826484	0.06683214	1.0387025	1.049482	0.44843736	0.43564552	0.0253131	0.025438279
20〇〇/〇〇/〇〇 10:27:10	0.07886149	0.06374172	1.0427829	1.0495228	0.4617904	0.43128288	0.0197062	0.018964142
20〇〇/〇〇/〇〇 10:27:11	0.09803399	0.07066488	1.0379735	1.0491341	0.43710756	0.43222567	0.01900212	0.016784705

Supplementary Figure 1 Information of the eight parameters and 9-s epoch in this study. Avg and cv denote average and coefficient variation, respectively. The red line square shows an example of the eight parameters. The blue line square depicts an example of the 9-s epoch.
