# Supplementary material for: Detection of Generalized Tonic–Clonic Seizures in Dogs With a Seizure Detection System Established Using Acceleration Data and the Mahalanobis Distance: A Preliminary Study
Source: Front Vet Sci. 2022 Apr 28;9:848604. doi: 10.3389/fvets.2022.848604 (PMC9097225; doi:10.3389/fvets.2022.848604)
Supplement: Supplementary file 5 [file Image_2.pdf]

Time	X-axis avg (g)	Y-axis avg (g)	Z-axis avg (g)	Resultant avg (g)	X-axis cv (g)	Y-axis cv (g)	Z-axis cv (g)	Resultant cv (g)
20〇〇/〇〇/〇〇 10:27:00	0.23585667	0.25572115	0.94563264	1.0554526	0.42863384	0.58072174	0.08409472	0.067146495
20〇〇/〇〇/〇〇 10:27:01	0.23982164	0.26510945	0.9422667	1.0546426	0.47810358	0.6263182	0.0922258	0.07586935
20〇〇/〇〇/〇〇 10:27:02	0.22422282	0.26760256	0.9432904	1.0540118	0.5368596	0.6315607	0.09436157	0.07830152
20〇〇/〇〇/〇〇 10:27:03	0.22201782	0.26788983	0.94498485	1.0550516	0.49430355	0.59799325	0.0875614	0.07175192
20〇〇/〇〇/〇〇 10:27:04	0.189128	0.26096818	0.9555981	1.0544344	0.46688333	0.49996412	0.07735945	0.06183452
20〇〇/〇〇/〇〇 10:27:05	0.17444256	0.2227722	0.9676049	1.0505434	0.37277424	0.4631078	0.05986796	0.047089994
20〇〇/〇〇/〇〇 10:27:06	0.16980699	0.15381253	0.9947251	1.0487041	0.3324536	0.45870128	0.04630764	0.038756315
20〇〇/〇〇/〇〇 10:27:07	0.16140653	0.09402873	1.0179272	1.0486298	0.34499964	0.4885503	0.03693672	0.034193344
20〇〇/〇〇/〇〇 10:27:08	0.13863334	0.06506795	1.0285888	1.0485132	0.40688527	0.48714215	0.03098419	0.03054409
20〇〇/〇〇/〇〇 10:27:09	0.09826484	0.06683214	1.0387025	1.049482	0.44843736	0.43564552	0.0253131	0.025438279
20〇〇/〇〇/〇〇 10:27:10	0.07886149	0.06374172	1.0427829	1.0495228	0.4617904	0.43128288	0.0197062	0.018964142
20〇〇/〇〇/〇〇 10:27:11	0.09803399	0.07066488	1.0379735	1.0491341	0.43710756	0.43222567	0.01900212	0.016784705

Test dataset 1
Test dataset 2
Test dataset 3
.
.
.
.
.
.
.
.
.

Supplementary Figure 2 How to extract the nonepileptic activity test dataset. All recorded data were divided into 9-s epochs in the test dataset, which were created repeatedly by shifting the beginning of the 9-s epoch by 1 s. The number of a test dataset generated from the recorded data had the mathematical rule. For instance, if there were 10s recorded data, two 9-s epoch test datasets were generated. If there were 11s data, the number of test datasets would be three. Thus, the number of test datasets will be “the total recorded seconds minus eight”.
