# Supplementary material for: Detection of Generalized Tonic–Clonic Seizures in Dogs With a Seizure Detection System Established Using Acceleration Data and the Mahalanobis Distance: A Preliminary Study
Source: Front Vet Sci. 2022 Apr 28;9:848604. doi: 10.3389/fvets.2022.848604 (PMC9097225; doi:10.3389/fvets.2022.848604)
Supplement: Supplementary file 6 [file Image_3.pdf]

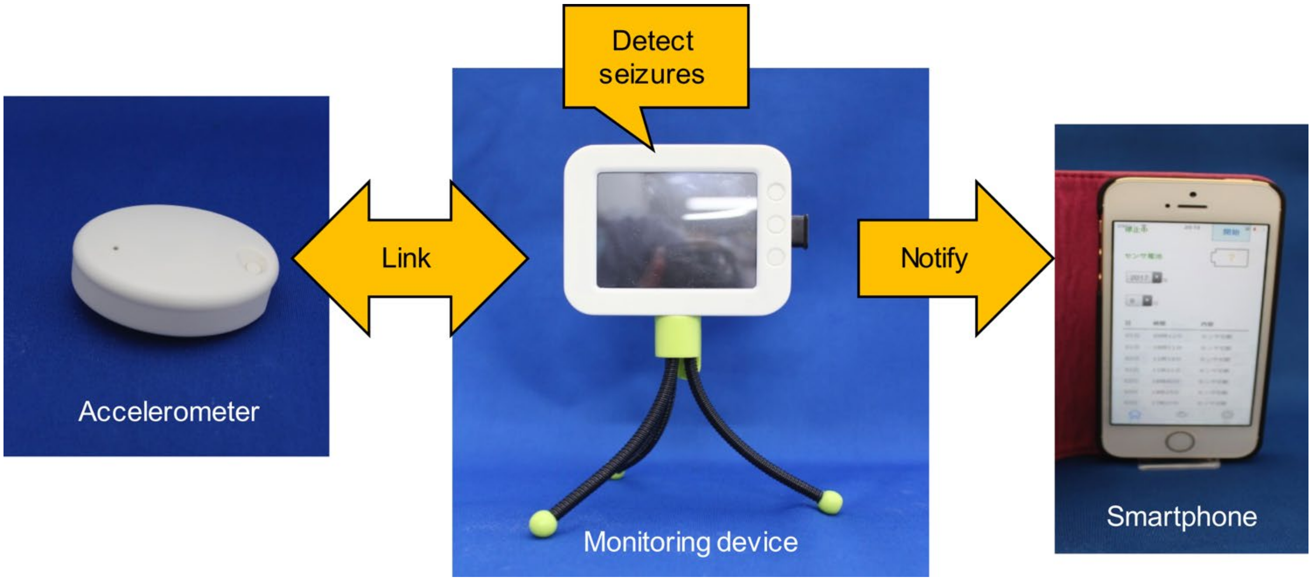

Supplementary Figure 3. Prototype of the seizure detection system (SDS). The SDS comprises a wireless three-axis accelerometer (width: 40 mm, height: 60 mm, depth: 15 mm, and weight: 25 g), monitoring device, and smartphone. The monitoring device implements the seizure detection algorithm and detects seizures with the acceleration data sent by the accelerometer. When the monitoring device identifies a seizure, it sends a notification to the smartphone.
